# Sextuple Tumors in Head and Neck Area: Evidence of Field Cancerization

**DOI:** 10.1155/2018/8428395

**Published:** 2018-08-15

**Authors:** Carla Bento Nelem-Colturato, Patrícia Maluf Cury, Thiago Machado Pereira, Isabelle Silva Cosso, Kellin Pivato, Luiz Evaristo Ricci Volpato, Alvaro Henrique Borges

**Affiliations:** ^1^Health and Sports Sciences Center, Federal University of Acre, Rio Branco, Acre, Brazil; ^2^Department of Pathology, The Ceres College School of Medicine (FACERES), São José do Rio Preto, São Paulo, Brazil; ^3^Department of Oral Sciences, University of Cuiaba, Cuiaba, Mato Grosso, Brazil

## Abstract

**Background:**

Field cancerization is a phenomenon in which prolonged exposure to carcinogens induces changes throughout the epithelium leaving the field ready for the appearance of premalignant or malignant lesions. These alterations can promote the development of multiple carcinomas and explain the appearance of recurrences and second primary tumors. The objective of this study was to report the case of a patient who developed six oral cavity tumors in five years of treatment and, also, demonstrate the immunohistochemical changes for p53 and Ki-67, routinely used to assess dysplasic regions.

**Case Report:**

When altered, p53 and Ki-67 suggest the presence of field cancers, an area with genetically altered cells, presenting a high risk of developing premalignant and malignant lesions. This phenomenon explains the recurrence of malignant neoplasms after tumor resections.

**Conclusion:**

In addition, early identification of potentially malignant lesions in cases of second primary tumors is essential for effective treatment and patient survival, which usually have an unwelcoming prognosis.

## 1. Background

The classic study of Slaughter et al. [1] proposed the concept of field cancerization as alterations, caused by carcinogenic agent, predisposing the development of cancer and explaining the development of multiple primary tumors and locally recurrent cancer in the upper aerodigestive tract.

The mutation of the p53 gene, often found in human cancers, is associated with smoking. Furthermore, mutations can be found in dysplasic lesions, indicating the genetic modification, which may precede the advent of malignancy [2, 3]. The Ki-67 antigen is an important protein on cell division process, correlated to the presence of sever epithelial dysplasia. Thus, p53 and Ki-67 are routinely field cancerization markers used to assess dysplastic regions [2, 4].

This paper reports the case of a patient who has developed six malignant tumors of the oral cavity in five years showing immunoreactivity for p53 and Ki-67 suggesting presence of field cancerization.

## 2. Case Report

A 63-year-old Caucasian male patient sought an otorhinolaryngology treatment in São José do Rio Preto, Brazil, complaining of continuous hoarseness. Patient reported being smoker and alcoholic for 40 years and having stopped smoking for five years and denied any systemic or local diseases. Clinical examination showed lesions in the piriform sinus during nasofibroscopy. Lesions were biopsied and the pathological diagnosis was moderately differentiated and invasive squamous cell carcinoma (SCC). Within a month, a laryngectomy was performed with a selective right cervical dissection followed by radiotherapy.

**Figure 1:**
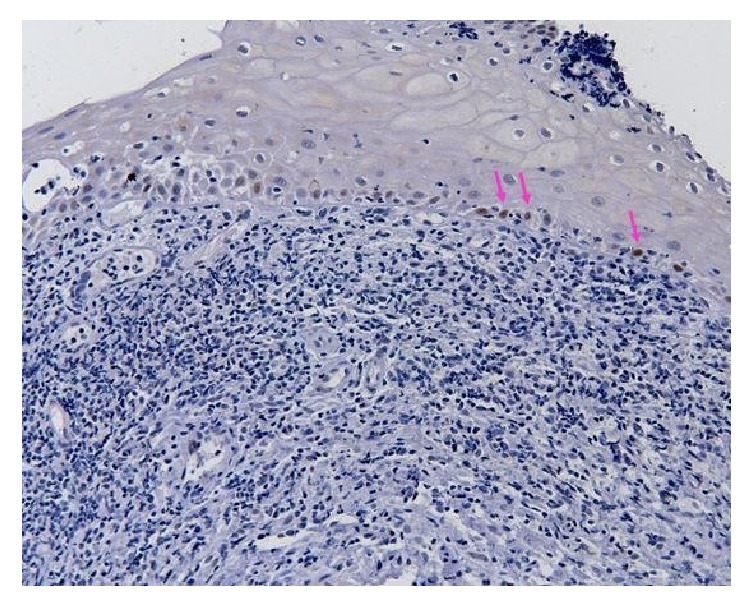
Normal region in the tumor margin. Arrows mark the immunoexpression of p53 in some focal areas in the basal layer with weak nuclear staining (200x Magnification).

**Figure 2:**
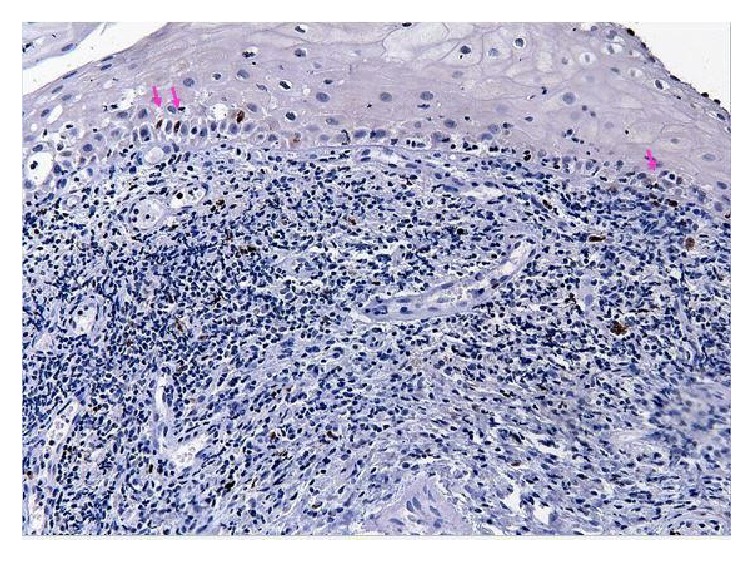
Normal region adjacent to the tumor. Arrows indicate positivity for Ki-67 in some focal areas with poor nuclear marking at the basal layer (200x Magnification).

At the first routine monitoring, 12 months later, patient presented a vegetating lesion on soft palate, diagnosed as moderately differentiated, and invasive SCC, a second neoplasm. The excision of the soft palate and complementary radiotherapy were performed. Twenty-four months after the first diagnostic, patient presented erythematous lesions on the soft palate and left tonsillar pillar, both identified as moderately differentiated SCC, third and fourth malignant tumors. Thirty-six months after the first diagnostic, patient had an ulcerative-infiltrative lesion in right tonsillar pillar, diagnosed as SCC, which is the fifth malignant tumor. Forty-six months after the diagnostic, the first malignancy the patient developed symptomatic lesions in base of tongue was diagnosed as nonspecific chronic glossitis. The tissue adjacent to the lesion was evaluated with immunohistochemical staining for p53 (Figure 1) with some focal areas in the basal and suprabasal layer with weak nuclear staining and Ki-67 (Figure 2) with the positivity of basal and suprabasal layer.

**Figure 3:**
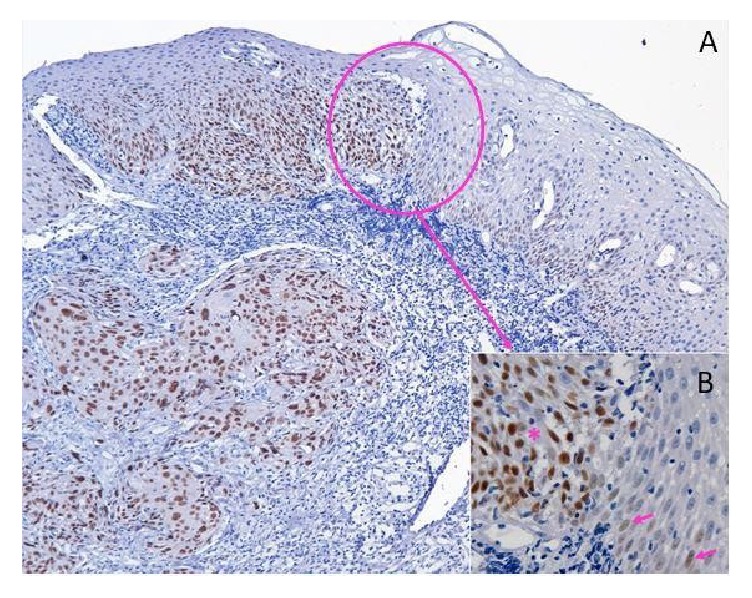
(A) Increased immunoreactivity of the p53 protein from the adjacent region towards the tumor region (less magnification); (B) transition area. Arrows indicate marking of nuclei to p53 in a region adjacent to the tumor; asterisk marks tumor region (highest magnification).

**Figure 4:**
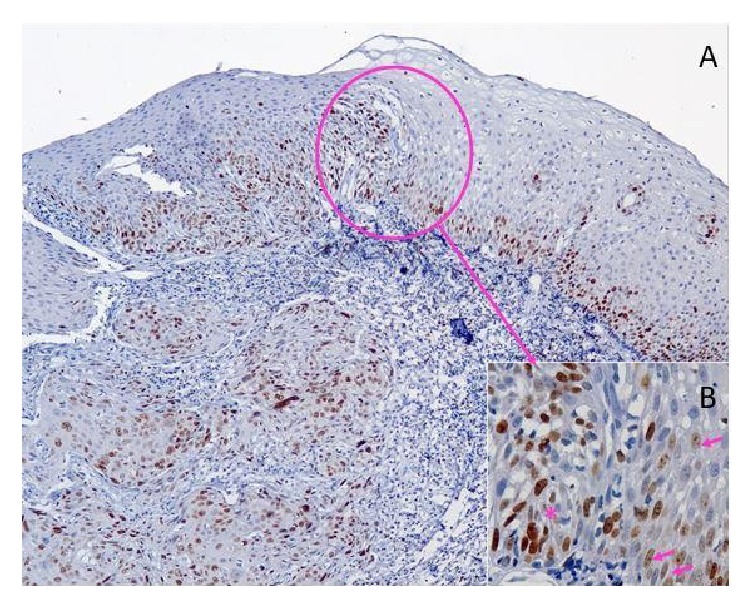
(A) Strong and diffuse immunoreactivity for Ki-67 in the region adjacent to the tumor (less magnification); (B) transition area. Arrows indicate nucleation markers for Ki-67 in the region adjacent to the tumor; asterisk marks tumor region (highest magnification).

A month later, another surgery was executed to remove a lesion located in the uvula, also diagnosed as moderately differentiated SCC, being the sixth malignancy. Likewise, immunohistochemical investigation was performed for p53 and Ki-67, contiguous to the lesion of the uvula and all lesions that preceded it. The tissue adjacent to the uvula expressed diffuse immunoreactivity for p53 (Figure 3) and Ki-67 (Figure 4) with strong nuclear staining in the basal and suprabasal layer. None of the neoplasms was accompanied by lymph node metastases. At the patient's return, 60 months after the first diagnostic, there was no evidence of other malignancies.

## 3. Discussion

The field cancerization is a phenomenon wherein prolonged exposure to carcinogens induces changes across the epithelium leaving the field suitable for malignant transformation [1]. However, the exposure to such agents, especially tobacco, results in a multifocal genetic transformation that may result in the development of multiple carcinomas [3, 5–7].

This may explain the recurrence and second primary tumors (SPT) development [3, 5–7]. Hong et al. [8] defined SPT: (1) each tumor must be geographically separate and distinct, and (2) the possibility that SPT is a metastasis or local recurrence must be excluded, in addition to multiple primary tumors as triple and quadruple [5, 9] and quintuple [9].

Tobacco and alcohol are carcinogens that act as synergists, increasing their deleterious effects on the emergence of squamous cell carcinoma [7, 10] and SPT [11]. The prognosis of patients with SPT is unwelcoming. Thus, the best strategy is prevention and early diagnosis especially in potentially malignant lesions [11].

In this case report, the patient presented three second synchronous primary tumors (simultaneous or within the range of less than six months after tumor diagnosis), three metachronous (diagnosed after six months), and two nonmalignant proliferative lesions. Several authors conclude that there is a correlation between increased risk of patients developing SPT and field cancerization changes [3, 7, 12]. Other studies have reported that genetic alterations demonstrated in the immunohistochemistry for p53 in nondysplastic lesions were premature events in carcinogenesis [3, 4, 6]. In this study, a strong and diffuse positivity for p53 was observed in tissues adjacent to squamous cell carcinomas, but poor marking in nonneoplastic lesions.

The occurrence of tumors in the same anatomical region (mesopharynx) with the same histological appearance (SCC) could have been due to a recurrence of the primary tumor rather than six separate entities. What differentiates the two situations is that, histologically, when the distance between the tumors is smaller than 2 cm, it is considered as a local recurrence, and when the distance is greater than 2 cm, it is considered as a second primary tumor [13]. In the present case, the distances of the anatomical sites were superior to 2 cm.

The presence of the phenomenon of cancer cell dormancy in the background of frequent recurrence of oropharyngeal cancers could also be suggested. However, in the presence of cancer cell dormancy, the cell proliferation markers (Ki-67 and Cyclin D1) decrease and increase p53 [14]. In the present case there was no loss of Ki-67 expression.

As the findings of Reddy et al. [15], it was observed in this case that basal and suprabasal layers were positive for p53 in areas adjacent to carcinomas, suggesting that field changes could increase the positivity in basal and suprabasal layers. An increased positivity for p53 due to tobacco use was reported. In the case described, the patient was a tobacco user for 40 years.

The basal layer of the oral epithelium is a cell proliferation region while the suprabasal layer is responsible for cellular maturation. The presence of mitosis in the basal layer should be considered as a reason for attention [16]. The Ki-67 antigen detects the presence of proliferative activity and, therefore, in the epithelial dysplasia, exhibits positive increase derived from the change of the proliferative activity of the suprabasal layer and the severity of epithelial dysplasia increase [16]. In the sample collected from the tongue base, positivity for Ki-67 increased as the severity of dysplasia progressed, being positive in the basal and suprabasal layer in the area that showed mild to moderate dysplasia, and the region with moderate to severe dysplasia got greater positivity, covering the lower and middle thirds, corroborating the study of Viswanathan et al. [17]. The presented case showed more positivity for Ki-67 in the area adjacent to carcinomas than in the areas adjacent to nonneoplastic lesions.

In addition, combined p53 and Ki-67 immunohistochemical expression could represent a simple and inexpensive way to use molecular markers for early detection of potentially malignant lesions with risk of oral cancer [4] and for detection of oral field cancers [18].

## 4. Conclusions

This report confirms the importance of immunohistochemistry evaluation for p53 and Ki-67 for the search of field cancerization and therefore its importance in monitoring specific sites for early diagnosis and consequently better prognosis. Knowing that these patients are likely to develop second primary tumors, health care professionals should monitor them thoughtfully and cautiously. Furthermore, literature presents scarce case reports with six primary tumors.

## References

[1] Slaughter D. P., Southwick H. W., Smejkal W. (1953). Field cancerization in oral stratified squamous epithelium; clinical implications of multicentric origins. *Cancer*.

[2] Rivera C., Venegas B. (2014). Histological and molecular aspects of oral squamous cell carcinoma. *Oncology Letters*.

[3] Mohan M., Jagannathan N. (2014). Oral field cancerization: an update on current concepts. *Oncology Reviews*.

[4] Gissi D. B., Gabusi A., Servidio D., Cervellati F., Montebugnoli L. (2015). Predictive role of p53 protein as a single marker or associated with ki67 antigen in oral leukoplakia: A retrospective longitudinal study. *The Open Dentistry Journal *.

[5] Németh Z., Czigner J., Iván L., Ujpál M., Barabás J., Szabó G. (2002). Quadruple cancer, including triple cancers in the head and neck region. *Neoplasma*.

[6] Simple M., Suresh A., Das D., Kuriakose M. A. (2015). Cancer stem cells and field cancerization of Oral squamous cell carcinoma. *Oral Oncology*.

[7] Hedberg M. L., Goh G., Chiosea S. I. (2016). Genetic landscape of metastatic and recurrent head and neck squamous cell carcinoma. *The Journal of Clinical Investigation*.

[8] Hong W. K., Lippman S. M., Itri L. M. (1990). Prevention of second primary tumors with isotretinoin in squamous-cell carcinoma of the head and neck. *The New England Journal of Medicine*.

[9] Martin-Granizo R., Naval L., Castro P., Goizueta C., Munoz M. (1997). Quintuple cancers: Report of a case with triple cancers in the head and neck. *Journal of Cranio-Maxillo-Facial Surgery*.

[10] Curado M. P., Boyle P. (2013). Epidemiology of head and neck squamous cell carcinoma not related to tobacco or alcohol. *Current Opinion in Oncology*.

[11] Priante A. V. M., Castilho E. C., Kowalski L. P. (2011). Second primary tumors in patients with head and neck cancer. *Current Oncology Reports*.

[12] Singh P. K., Bogra J., Chandra G. (2015). Association of TNF-*α* (-238 and -308) promoter polymorphisms with susceptibility of oral squamous cell carcinoma in North Indian population. *Cancer Biomarkers*.

[13] Clark D. J., Mao L. (2017). Understanding the Surgical Margin: A Molecular Assessment. *Oral and Maxillofacial Surgery Clinics of North America*.

[14] Gao X.-L., Zhang M., Tang Y.-L., Liang X.-H. (2017). Cancer cell dormancy: Mechanisms and implications of cancer recurrence and metastasis. *OncoTargets and Therapy*.

[15] Reddy V. M., Kamath A., Radhakrishnan R. A. (2012). P53 immunoprofiling of potentially malignant oral disorders: A case series analysis. *Indian Journal of Cancer*.

[16] Birajdar S. S., Radhika M. B., Paremala K., Sudhakara M., Soumya M., Gadivan M. (2014). Expression of Ki-67 in normal oral epithelium, leukoplakic oral epithelium and oral squamous cell carcinoma. *Journal of Oral and Maxillofacial Pathology*.

[17] Viswanathan V., Juluri R., Goel S., Madan J., Mitra S. K., Gopalakrishnan D. (2015). Apoptotic index and proliferative index in premalignant and malignant squamous cell lesions of the oral cavity. *Journal of International Oral Health*.

[18] Monti-Hughes A., Aromando R. F., Pérez M. A., Schwint A. E., Itoiz M. E. (2015). The hamster cheek pouch model for field cancerization studies. *Periodontology 2000*.

